# Iterative hard thresholding in genome-wide association studies: Generalized linear models, prior weights, and double sparsity

**DOI:** 10.1093/gigascience/giaa044

**Published:** 2020-06-03

**Authors:** Benjamin B Chu, Kevin L Keys, Christopher A German, Hua Zhou, Jin J Zhou, Eric M Sobel, Janet S Sinsheimer, Kenneth Lange

**Affiliations:** 1 Department of Computational Medicine, University of California, Los Angeles, 621 Charles E Young Dr S, Los Angeles, CA, 90095, USA; 2 Department of Medicine, University of California, San Francisco, 1701 Divisadero St, San Francisco, CA, 94115, USA; 3 Berkeley Institute of Data Science, University of California, Berkeley, 190 Doe Library, Berkeley, CA 94720, USA; 4 Department of Biostatistics, University of California, Los Angeles, 650 Charles E Young Dr S, Los Angeles, CA, 90095, USA; 5 Division of Epidemiology and Biostatistics, University of Arizona, 1295 N. Martin Ave. Tucson, AZ, 85724, USA; 6 Department of Human Genetics, University of California, Los Angeles, 695 Charles E Young Dr S, Los Angeles, CA, 90095 USA

**Keywords:** GWAS, multiple regression, high dimensional inference, iterative hard thresholding, biobank

## Abstract

**Background:**

Consecutive testing of single nucleotide polymorphisms (SNPs) is usually employed to identify genetic variants associated with complex traits. Ideally one should model all covariates in unison, but most existing analysis methods for genome-wide association studies (GWAS) perform only univariate regression.

**Results:**

We extend and efficiently implement iterative hard thresholding (IHT) for multiple regression, treating all SNPs simultaneously. Our extensions accommodate generalized linear models, prior information on genetic variants, and grouping of variants. In our simulations, IHT recovers up to 30% more true predictors than SNP-by-SNP association testing and exhibits a 2–3 orders of magnitude decrease in false-positive rates compared with lasso regression. We also test IHT on the UK Biobank hypertension phenotypes and the Northern Finland Birth Cohort of 1966 cardiovascular phenotypes. We find that IHT scales to the large datasets of contemporary human genetics and recovers the plausible genetic variants identified by previous studies.

**Conclusions:**

Our real data analysis and simulation studies suggest that IHT can (i) recover highly correlated predictors, (ii) avoid over-fitting, (iii) deliver better true-positive and false-positive rates than either marginal testing or lasso regression, (iv) recover unbiased regression coefficients, (v) exploit prior information and group-sparsity, and (vi) be used with biobank-sized datasets. Although these advances are studied for genome-wide association studies inference, our extensions are pertinent to other regression problems with large numbers of predictors.

## Introduction

In genome-wide association studies (GWAS), modern genotyping technology coupled with imputation algorithms can produce an *n* × *p* genotype matrix **X** with *n* ≈ 10^6^ subjects and *p* ≈ 10^7^ genetic predictors [1, 2]. Datasets of this size require hundreds of gigabytes of disk space to store in compressed form. Decompressing data to floating point numbers for statistical analyses leads to matrices too large to fit into standard computer memory. The computational burden of dealing with massive GWAS datasets limits statistical analysis and interpretation. This article discusses and extends a class of algorithms capable of meeting the challenge of multiple-regression models scaled to the size of modern GWAS datasets.

Traditionally, GWAS analysis has focused on SNP-by-SNP (single-nucleotide polymorphism) association testing [1, 3], with a *P*-value computed for each SNP via linear regression. This approach enjoys the advantages of simplicity, interpretability, and a low computational complexity of $\mathcal {O}(np)$. Furthermore, marginal linear regressions make efficient use of computer memory because computations are carried out on genotype vectors one at a time, as opposed to running on the full genotype matrix in multiple regression. Some authors further increase association power by reframing GWAS as a linear mixed-model problem and proceeding with variance component selection [4, 5]. These advances remain within the scope of marginal analysis.

Despite their numerous successes [2], marginal regression is less than ideal for GWAS. It implicitly assumes that all SNPs have independent effects. In contrast, multiple regression can in principle model the effect of all SNPs simultaneously. This approach captures the biology behind GWAS more realistically because traits are usually determined by multiple SNPs acting in unison. Marginal regression selects associated SNPs 1 by 1 on the basis of a pre-set threshold. Given the stringency of the *P*-value threshold, marginal regression can miss many causal SNPs with low effect sizes. As a result, heritability is underestimated. When *p* ≫ *n*, one usually assumes that the number of variants *k* associated with a complex trait is much smaller than *n*. If this is true, we can expect multiple-regression models to perform better because they (i) offer better outlier detection [6] and better prediction, (ii) account for the correlations among SNPs, and (iii) allow investigators to model interactions. Of course, these advantages are predicated on finding the truly associated SNPs.

Adding penalties to the loss function is one way of achieving parsimony in multiple regression. The lasso [7, 8] is the most popular model selection device in current use. The lasso model selects non-zero parameters by minimizing the criterion

$$\begin{eqnarray*}
f(\boldsymbol{\beta }) & = & \ell (\boldsymbol{\mathrm {\beta} }) + \lambda \Vert \boldsymbol{\beta }\Vert _1,
\end{eqnarray*}$$

where $\ell (\boldsymbol{\beta })$ is a convex loss, λ is a sparsity tuning constant, and $\Vert \boldsymbol{\beta }\Vert _1 = \sum _j |\beta _j|$ is the ℓ_1_ norm of the parameters. The lasso has the virtues of preserving convexity and driving most parameter estimates to 0. Minimization can be conducted efficiently via cyclic coordinate descent [9, 10]. The magnitude of the nonzero tuning constant λ determines the number of predictors selected.

Despite its widespread use, the lasso penalty has some drawbacks. First, the ℓ_1_ penalty tends to shrink parameters toward 0, sometimes severely so. Second, λ must be tuned to achieve a given model size. Third, λ is chosen by cross-validation, a costly procedure. Fourth and most importantly, the shrinkage caused by the penalty leaves a lot of unexplained trait variance, which tends to encourage too many false-positive results to enter the model ultimately identified by cross-validation.

Inflated false-positive rates can be mitigated by substituting nonconvex penalties for the ℓ_1_ penalty. For example, the minimax concave penalty (MCP) [11]

$$\begin{eqnarray*}
\lambda p(\beta _j) & = & \lambda \int _0^{|\beta _j|} \left(1- \frac{s}{\lambda \gamma }\right)_+ ds
\end{eqnarray*}$$

starts out at β_*j*_ = 0 with slope λ and gradually transitions to a slope of 0 at β_*j*_ = λγ. With minor adjustments, the coordinate descent algorithm for the lasso carries over to MCP penalized regression [12, 13]. Model selection is achieved without severe shrinkage, and inference in GWAS improves [14]. However, in our experience its false-negative rate is considerably higher than iterative hard thresholding (IHT)’s rate [15]. A second remedy for the lasso, stability selection, weeds out false-positive results by looking for consistent predictor selection across random halves of the data [16]. However, it is known to be under-powered for GWAS compared to standard univariate selection [17].

**Figure 1:**
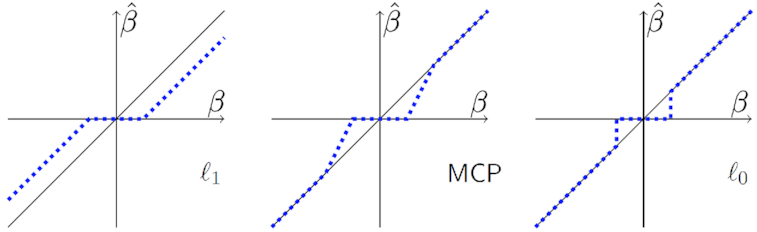
The ℓ_0_ quasinorm of IHT enforces sparsity without shrinkage. The estimated effect size (dashed line) is plotted against its true value (diagonal line) for ℓ_1_, MPC, and ℓ_0_ penalties.

In contrast, IHT minimizes a loss $\ell (\boldsymbol{\beta })$ subject to the nonconvex sparsity constraint $\Vert \boldsymbol{\beta }\Vert _0 \le k$, where $\Vert \boldsymbol{\beta }\Vert _0$ counts the number of non-zero components of $\boldsymbol{\beta }$ [18–20]. Fig. 1 explains graphically how the ℓ_0_ penalty of IHT reduces the bias of the selected parameters compared to ℓ_1_ and MPC penalties. In the figure λ, γ, and *k* are chosen so that the same range of β values are sent to zero. To its detriment, the lasso penalty shrinks all β’s, no matter how large their absolute values. The nonconvex MCP penalty avoids shrinkage for large β’s but exerts shrinkage for intermediate β’s. IHT, which is both nonconvex and discontinuous, avoids shrinkage altogether. For GWAS, the sparsity model-size constant *k* also has a simpler and more intuitive interpretation than the lasso tuning constant λ. Finally, both false-positive and false-negative rates are well controlled. Balanced against these advantages is the loss of convexity in optimization and concomitant loss of computational efficiency. In practice, the computational barriers are surmountable and are compensated by the excellent results delivered by IHT in high-dimensional regression problems such as multiple GWAS regression.

This article has 4 interrelated goals. First, we extend IHT to generalized linear models (GLM). These models encompass most of applied statistics. Previous IHT algorithms focused on normal or logistic sparse regression scenarios. Our software can also perform sparse regression under Poisson and negative binomial response distributions and can be easily extended to other GLM distributions as needed. The key to our extension is the derivation of a nearly optimal step size *s* for improving the loglikelihood at each iteration. Second, we introduce doubly sparse regression to IHT. Previous authors have considered group sparsity [21]. The latter tactic limits the number of groups selected. It is also useful to limit the number of predictors selected per group. Double sparsity strikes a compromise that encourages selection of correlated causative variants in linkage disequilibrium (LD). Notably, this technique generalizes group-IHT. Third, we demonstrate how to incorporate predetermined SNP weights in IHT. Our simple and interpretable weighting option allows users to introduce prior knowledge into sparse projection. Thus, one can favor predictors whose association to the response is supported by external evidence. Fourth, we present MendelIHT.jl: a scalable, open source, and user-friendly software for IHT in the high-performance programming language Julia [22].

## Model Development

This section sketches our extensions of IHT.

### IHT background

IHT was originally formulated for sparse signal reconstruction, which is framed as sparse linear least-squares regression. In classical linear regression, we are given an *n* × *p* design matrix **X** and a corresponding *n*-component response vector **y**. We then postulate that **y** has mean ${\rm E}({\bf y})={\bf X}\boldsymbol{\beta }$ and that the residual vector ${\bf y}-{\bf X}\boldsymbol{\beta }$ has independent Gaussian components with a common variance. The parameter (regression coefficient) vector $\boldsymbol{\beta }$ is estimated by minimizing the sum of squares $f(\boldsymbol{\beta })= (1/2)\Vert {\bf y} - {\bf X} \boldsymbol{\beta }\Vert _2^2$. The solution to this problem is known as the ordinary least-squares estimator and can be written explicitly as $\hat{\boldsymbol{\beta }} = ({\bf X}^t {\bf X})^{-1}{\bf X}^t {\bf y}$, provided the problem is overdetermined (*n* > *p*). This paradigm breaks down in the high-dimensional regime *n* ≪ *p*, where the parameter vector $\boldsymbol{\beta }$ is underdetermined. In the spirit of parsimony, IHT seeks a sparse version of $\boldsymbol{\beta }$ that gives a good fit to the data. This is accomplished by minimizing $f(\boldsymbol{\beta })$ subject to $\Vert \boldsymbol{\beta }\Vert _0 \le k$ for a small value of *k*, where ‖ · ‖_0_ counts the number of nonzero entries of a vector. The optimization problem is formally:

(1) $$\begin{eqnarray*}
\min \frac{1}{2}||{\bf y} - {\bf X}\boldsymbol{\beta }||^2_2 \quad \text{subject to } ||\boldsymbol{\beta }||_0 \le k.
\end{eqnarray*}$$

IHT abandons the explicit formula for $\hat{\boldsymbol{\beta }}$ because it fails to respect sparsity and involves the numerically intractable matrix inverse (**X**^*t*^**X**)^−1^.

IHT combines 3 core ideas. The first is steepest descent. Elementary calculus tells us that the negative gradient −∇*f*(**x**) is the direction of steepest descent of $f(\boldsymbol{\beta })$ at **x**. First-order optimization methods like IHT define the next iterate in minimization by the formula $\boldsymbol{\beta }_{n+1}= \boldsymbol{\beta }_n + s_n {\bf v}_n$, where ${\bf v}_n = -\nabla f(\boldsymbol{\beta }_n)$ and *s_n_* > 0 is some optimally chosen step size. In the case of linear regression $-\nabla f(\boldsymbol{\beta })={\bf X}^t({\bf y}-{\bf X}\boldsymbol{\beta })$. To reduce the error at each iteration, the optimal step size *s_n_* can be selected by minimizing the second-order Taylor expansion

$$\begin{eqnarray*}
& & f(\boldsymbol{\beta }_n+s_n{\bf v}_n) \\
& = & f(\boldsymbol{\beta }_n)+s_n\nabla f(\boldsymbol{\beta }_n)^t{\bf v}_n +\frac{s_{n}^2}{2} {\bf v}_n^t d^2f(\boldsymbol{\beta }_n){\bf v}_n \\
& = & f(\boldsymbol{\beta }_n)-s_n \Vert \nabla f(\boldsymbol{\beta }_n)\Vert _2^2 +\frac{s_{n}^2}{2} \nabla f(\boldsymbol{\beta }_n)^t d^2f(\boldsymbol{\beta }_n)\nabla f(\boldsymbol{\beta }_n)
\end{eqnarray*}$$

with respect to *s_n_*. Here $d^2f(\boldsymbol{\beta }) = {\bf X}^t{\bf X}$ is the Hessian matrix of second partial derivatives. Because $f(\boldsymbol{\beta })$ is quadratic, the expansion is exact. Its minimum occurs at the step size

(2) \begin{eqnarray*} s_n & = & \frac{\Vert \nabla f(\boldsymbol{\beta }_n)\Vert _2^2}{\nabla f(\boldsymbol{\beta }_n)^t d^2f(\boldsymbol{\beta }_n)\nabla f(\boldsymbol{\beta }_n)}. \end{eqnarray*}

This formula summarizes the second core idea.

The third component of IHT involves projecting the steepest descent update $\boldsymbol{\beta }_n + s_n {\bf v}_n$ onto the sparsity set $S_k = \lbrace \boldsymbol{\beta } : \Vert \boldsymbol{\beta }\Vert _0 \le k\rbrace$. The relevant projection operator $P_{S_k}(\boldsymbol{\beta })$ sets all but the *k* largest entries of $\boldsymbol{\beta }$ in magnitude to 0. In summary, IHT solves problem (1) by updating the parameter vector $\boldsymbol{\beta }$ according to the recipe:

$$\begin{eqnarray*}
\boldsymbol{\beta }_{n+1} & = & P_{S_k}\left[\boldsymbol{\beta }_n - s_n \nabla f(\boldsymbol{\beta }_n)\right]
\end{eqnarray*}$$

with the step size given by formula (2).

An optional debiasing step can be added to improve parameter estimates. This involves replacing $\boldsymbol{\beta }_{n+1}$ by the exact minimum point of $f(\boldsymbol{\beta })$ in the subspace defined by the support $\lbrace j:\boldsymbol{\beta }_{n+1,j} \ne 0\rbrace$ of $\boldsymbol{\beta }_{n+1}$. Debiasing is efficient because it solves a low-dimensional problem. Several versions of hard-thresholding algorithms have been proposed in the signal-processing literature. The first of these, NIHT [20], omits debaising. The rest, HTP [23], GraHTP [24], and CoSaMp [25], offer debiasing.

### IHT for generalized linear models

A GLM involves responses *y* following a natural exponential distribution with density in the canonical form

$$\begin{eqnarray*}
f(y \mid \theta , \phi ) & = & \exp \left[ \frac{y \theta - b(\theta )}{a(\phi )} + c(y, \phi ) \right],
\end{eqnarray*}$$

where *y* is the data, θ is the natural parameter, ϕ > 0 is the scale (dispersion), and *a*(ϕ), *b*(θ), and *c*(*y*, ϕ) are known functions that vary depending on the distribution [26, 27]. Simple calculations show that *y* has mean μ = *b*′(θ) and variance σ^2^ = *b*″(θ)*a*(ϕ); accordingly, σ^2^ is a function of μ. Table 1 summarizes the mean domains and variances of a few common exponential families. Covariates enter GLM modeling through an inverse link representation $\mu = g({\bf x}^t\boldsymbol{\beta })$, where **x** is a vector of covariates (predictors) and $\boldsymbol{\beta }$ is vector of regression coefficients (parameters). In statistical practice, data arrive as a sample of independent responses *y*_1_, …, *y_m_* with different covariate vectors **x**_1_, …, **x**_*m*_. To put each predictor on an equal footing, each should be standardized to have mean 0 and variance 1. Including an additional intercept term is standard practice.

**Table 1:** Summary of mean domains and variances for common exponential distributions

Family	Mean domain	var(*y*)	*g*(*s*)
Normal	$\mathbb {R}$	ϕ^2^	1
Poisson	[0, ∞)	μ	*e* ^*s*^
Bernoulli	[0,1]	μ(1 − μ)	$e^s/\left(1+e^s\right)$
Gamma	[0, ∞)	μ^2^ϕ	*s* ^−1^
Inverse Gaussian	[0, ∞)	μ^3^ϕ	*s* ^−1/2^
Negative binomial	[0, ∞)	μ(μϕ + 1)	*e* ^*s*^

In GLM, $\boldsymbol{\mu } = g({\bf x}^t\boldsymbol{\beta })$ denotes the mean, $s = {\bf x}^t\boldsymbol{\beta }$ the linear responses, *g* is the inverse link function, and ϕ the dispersion. Except for the negative binomial, all inverse links are canonical.

If we assemble a design matrix **X** by stacking the row vectors ${\bf x}_i^t$, then we can calculate the loglikelihood, score, and expected information [26–29]

(3) \begin{eqnarray*} L(\boldsymbol{\beta }) &=& \sum \nolimits _{i=1}^n \left[\frac{y_i \theta _i - b_i(\theta _i)}{a_i(\phi _i)} + c(y_i, \phi _i)\right] \nonumber \\ \nabla L(\boldsymbol{\beta }) &=& \sum \nolimits _{i=1}^n (y_i -\mu _i) \frac{g^{\prime }({\bf x}_i^t\boldsymbol{\beta })}{\sigma _i^2}{\bf x}_i = {\bf X}^t{\bf W}_{\! 1}({\bf y} - \boldsymbol{\mu }) \nonumber \\ J(\boldsymbol{\beta }) &=& \sum \nolimits _{i=1}^n \frac{1}{\sigma _i^2}g^{\prime }({\bf x}_i^t\boldsymbol{\beta })^2 {\bf x}_i{\bf x}_i^t = {\bf X}^t {\bf W}_{\! 2} {\bf X} , \end{eqnarray*}

where **W**_1_ and **W**_2_ are two diagonal matrices. The second has positive diagonal entries; they coincide under the identity inverse link *g*(*s*) = *s*.

In the GLM version of IHT, we maximize *L*(β) [equivalent to minimizing $f(\boldsymbol{\beta })= -L(\boldsymbol{\beta })$] and substitute the expected information $J(\boldsymbol{\beta }_n) = {\rm E}[-d^2L(\boldsymbol{\beta }_n)]$ for $d^2f(\boldsymbol{\beta }_n)$ in formula (2). This translates into the following step size in GLM estimation:

(4) \begin{eqnarray*} s_n & = & \frac{\Vert \nabla L(\boldsymbol{\beta }_n)\Vert _2^2}{\nabla L(\boldsymbol{\beta }_n)^t J(\boldsymbol{\beta }_n)\nabla L(\boldsymbol{\beta }_n)}. \end{eqnarray*}

This substitution is a key ingredient of our extended IHT. It simplifies computations and guarantees that the step size is nonnegative.

### Doubly sparse projections

The effectiveness of group sparsity in penalized regression has been demonstrated in general [30, 31] and for GWAS [32] in particular. Group IHT [21] enforces group sparsity but does not enforce within-group sparsity. In GWAS, model selection is desired within groups as well to pinpoint causal SNPs. Furthermore, a concern in GWAS is that two causative SNPs can be highly correlated with each other due to LD. When sensible group information is available, doubly sparse IHT encourages the detection of causative yet correlated SNPs while enforcing sparsity within groups. Here we discuss how to carry out a doubly sparse projection that enforces both within- and between-group sparsity.

Suppose we divide the SNPs of a study into a collection *G* of nonoverlapping groups. Given a parameter vector $\boldsymbol{\beta }$ and a group *g* ∈ *G*, let $\boldsymbol{\beta }_{g}$ denote the components of $\boldsymbol{\beta }$ corresponding to the SNPs in *g*. Now suppose we want to select at most *j* groups and at most $\lambda _g \in \mathbb {Z}^+$ SNPs for each group *g*. In projecting $\boldsymbol{\beta }$, the component β_*i*_ is untouched for a selected SNP *i*. For an unselected SNP, β_*i*_ is reset to 0. By analogy with our earlier discussion, we can define a sparsity projection operator $P_{g}(\boldsymbol{\beta }_g)$ for each group *g*; $P_{g}(\boldsymbol{\beta }_g)$ selects the λ_*g*_ most prominent SNPs in group *g*. The potential reduction in the squared distance offered by group *g* is $r_g = \Vert \boldsymbol{\beta }_g\Vert _2^2 - \Vert P_{g}(\boldsymbol{\beta }_g)\Vert _2^2$. The *j* selected groups are determined by selecting the *j* largest values of *r_g_*. If desired, we can set the sparsity level λ*_g_*for each group high enough so that all SNPs in group *g* come into play. Thus, doubly sparse IHT generalizes group IHT. In Algorithm 1, we write $P(\boldsymbol{\beta })$ for the overall projection with the component projections $P_{g}(\boldsymbol{\beta }_g)$ on the *j* selected groups and projection to zero on the remaining groups.

### Prior weights in IHT

Zhou et al. [32] treat prior weights in penalized GWAS. Before calculating the lasso penalty, they multiply each component of the parameter vector $\boldsymbol{\beta }$ by a positive weight *w_i_*. We can do the same in IHT before projection. Thus, instead of projecting the steepest descent step $\boldsymbol{\beta } =\boldsymbol{\beta }_n+s_n {\bf v}_n$, we project the Hadamard (pointwise) product ${\bf w} \circ \boldsymbol{\beta }$ of $\boldsymbol{\beta }$ with a weight vector **w**. This produces a vector with a sensible support *S*. The next iterate $\boldsymbol{\beta }_{n+1}$ is defined to have support *S* and to be equal to $\boldsymbol{\beta }_n+s_n {\bf v}_n$ on *S*.

In GWAS, weights can and should be informed by prior biological knowledge. A simple scheme for choosing nonconstant weights relies on minor-allele frequencies (MAFs). For instance, Zhou et al. [33] assign SNP *i* with MAF *p_i_* the weight $w_i = 1/\left[2 p_i(1-p_i)\right]^{1/2}$. Giving rare SNPs greater weight in this fashion is most appropriate for traits under strong negative selection [34, 35]. Alternatively, our software permits users to assign weights geared to specific pathway and gene information.

de Lamare and Rodrigo [36] incorporate prior weights into IHT by adding an element-wise logarithm of a weight vector **q** before projection. The weight vector **q** is updated iteratively and requires 2 additional tuning constants that in practice are only obtained through cross-validation. Our weighting scheme is simpler, more computationally efficient, and more interpretable.

### Algorithm summary

The final algorithm combining doubly sparse projections, prior weight scaling, and debiasing is summarized in Algorithm 1.

**Algorithm 1:** Iterative hard thresholding

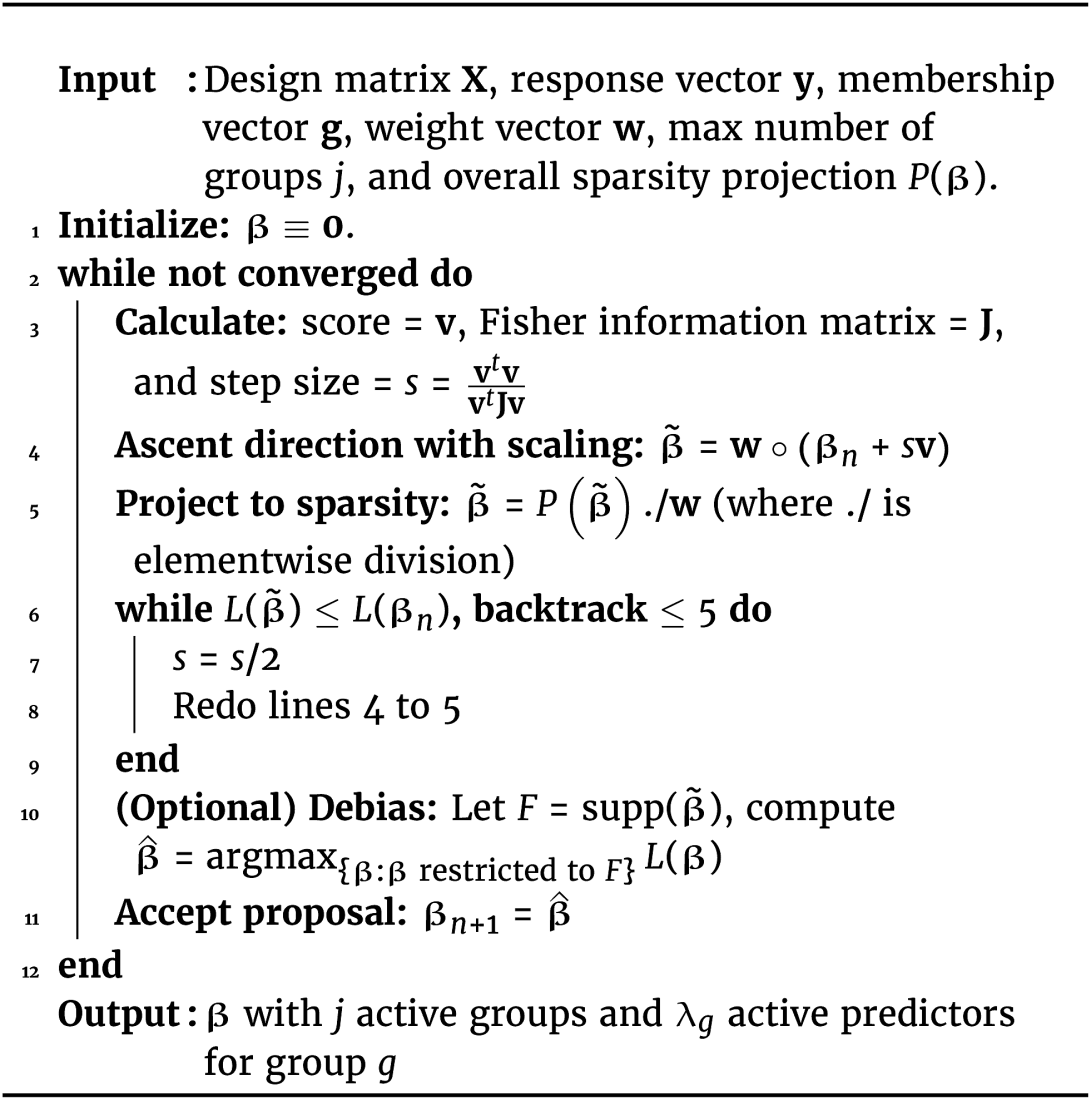

## Results

Readers can reproduce our results by accessing the software, documentation, and Jupyter notebooks on our Github page: https://github.com/OpenMendel/MendelIHT.jl

### Scalability of IHT

**Figure 2:**
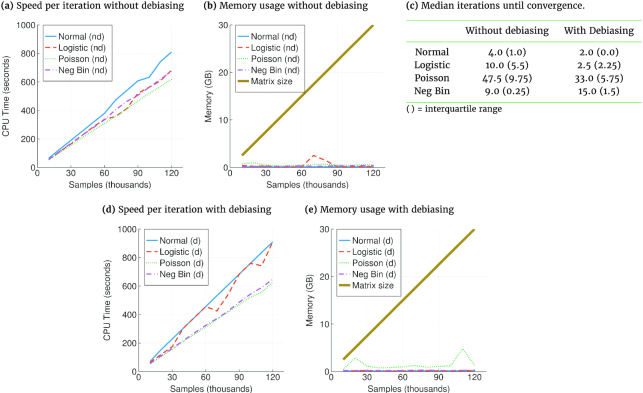
(a, d) Median Time per iteration scales linearly with data size. Speed is measured for compressed genotype files. On uncompressed data, all responses are roughly 10 times faster. (b, e) Median memory usage scales as ∼2*np* bits. Note memory for each response are usages in addition to loading the genotype matrix. Uncompressed data require 32 times more memory. (c) Debiasing reduces median iterations until convergence for all but negative binomial (Neg Bin) regression. Benchmarks were carried out on compressed data with 10^6^ SNPs and sample sizes ranging from 10,000 to 120,000. Hence, the largest matrix here requires 30 GB and can still fit into personal computer memories.

To test the scalability of our implementation, we ran IHT on *p* = 10^6^ SNPs for sample sizes *n* = 10,000, 20,000, ..., 120,000 with 5 independent replicates per *n*. All simulations rely on a true sparsity level of *k* = 10. Using a machine with 63 GB of RAM and a single 3.3-GHz Intel-E5-2670, Fig. 2 plots the IHT median CPU time per iteration, median iterations to convergence, and median memory usage under Gaussian, logistic, Poisson, and negative binomial models. The largest matrix simulated here is 30 GB in size and can still fit into our personal computer’s memory. Of course, it is possible to test even larger sample sizes using cloud or cluster resources, which are often needed in practice.

The formation of the vector $\boldsymbol{\mu }$ of predicted values requires only a limited number of nonzero regression coefficients. Consequently, the computational complexity of this phase of IHT is relatively light. In contrast, calculation of the Fisher score (gradient) and information (expected negative Hessian) depends on the entire genotype matrix **X**. Fortunately, each of the *np* entries of **X** can be compressed to 2 bits. Fig. 2b and e show that IHT memory demands beyond storing **X** never exceeded a few gigabytes. Fig. 2a and d show that IHT run time per iteration increases linearly in problem size *n*. Similarly, we expect increasing *p* will increase run time linearly because the bottleneck of IHT is the matrix-vector multiplication step in computing the gradient, which scales as *O*(*np*). Debiasing increases run time per iteration only slightly. Except for negative binomial responses, debiasing is effective in reducing the number of iterations required for convergence and hence overall run time.

### Cross-validation in model selection

**Figure 3:**
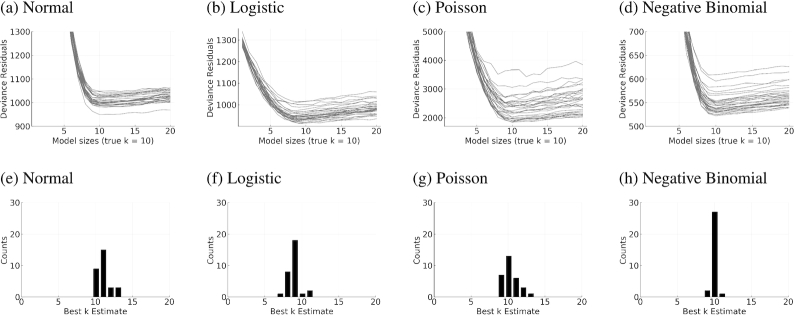
Five-fold cross-validation results are capable of identifying the true model size *k*_true_. (a–d) Deviance residuals of the testing set are minimized when the estimated model size $\hat{k} \approx k_{\rm true}$. Each line represents 1 simulation. (e–h) $\hat{k}$ is narrowly spread around *k*_true_ = 10.

In actual studies, the true number of genetic predictors *k*_true_ is unknown. This section investigates how *q*-fold cross-validation can determine the best model size on simulated data. Under normal, logistic, Poisson, and negative binomial models, we considered 50 different combinations of **X, y**, and $\boldsymbol{\beta }_{\rm true}$ with *k*_true_ = 10, *n* = 5,000 samples, and *p* = 50,000 SNPs fixed in all replicates. Here, *k*_true_ is chosen so that it is closer to our NFBC and UK Biobank results. On these datasets we conducted 5-fold cross-validation across 20 model sizes *k* ranging from 1 to 20. Fig. 3 plots deviance residuals on the holdout dataset for each of the 4 GLM responses (mean squared error in the case of normal responses) and the best estimate $\hat{k}$ of *k*_true_.

Fig. 3 shows that *k*_true_ can be effectively recovered by cross-validation. In general, prediction error starts off high where the proposed sparsity level *k* severely underestimates *k*_true_ and plateaus when *k*_true_ is reached (Fig. 3a–d). Furthermore, the estimated sparsity $\hat{k}$ for each run is narrowly centered around *k*_true_ = 10 (Fig. 3e and f). In fact, $|\hat{k} -k_{\rm true}|\le 4$ always holds. When $\hat{k}$ exceeds *k*_true_, the estimated regression coefficients for the false predictors tend to be very small. In other words, IHT is robust to overfitting, in contrast to lasso penalized regression. We see qualitatively similar results when *k*_true_ is large. This proved to be the case in our previous article [15] for Gaussian models with *k*_true_ ∈ {100, 200, 300}.

### Comparing IHT to lasso and marginal tests in model selection

Comparison of the true-positive and false-positive rates of IHT and its main competitors is revealing. For lasso regression we use the glmnet implementation of cyclic coordinate descent [9, 10, 37] (v2.0-16 implemented in R 3.5.2); for marginal testing we use the beta version of MendelGWAS [38]. As explained later, Poisson regression is supplemented by zero-inflated Poisson regression implemented under the pscl [39] (v1.5.2) package of R. Unfortunately, glmnet does not accommodate negative binomial regression. Because both glmnet and pscl operate on floating point numbers, we limit our comparisons to small problems with 1,000 subjects, 10,000 SNPs, 50 replicates, and *k* = 10 causal SNPs. IHT performs model selection by 3-fold cross-validation across model sizes ranging from 1 to 50. This range is generous enough to cover the models selected by lasso regression. We adjust for multiple testing in the marginal case by applying a *P*-value cut-off of 5 × 10^−6^.

Table 2 demonstrates that IHT achieves the best balance between maximizing true-positive results and minimizing false-positive results. IHT finds more true-positive results than marginal testing and almost as many as lasso regression. IHT also finds far fewer false-positive results than lasso regression. Poisson regression is exceptional in yielding an excessive number of false-positive results in marginal testing. A similar but less extreme trend is observed for lasso regression. The marginal false-positive rate is reduced by switching to zero-inflated Poisson regression. This alternative model is capable of handling overdispersion due to an excess of 0 values. Interestingly, IHT rescues the Poisson model by accurately capturing the simultaneous impact of multiple predictors.

**Table 2:** IHT achieves the best balance of false-positive and true-positive results compared to lasso and marginal (single-SNP) regression

Test	Normal	Logistic	Poisson	Neg Bin
IHT				
TP	8.84	6.28	7.20	8.88
FP	0.02	0.10	1.28	0.22
Lasso				
TP	9.52	8.16	9.28	NA
FP	31.26	45.76	102.24	NA
Marginal				
TP	7.18	5.76	9.04 (5.94)	5.98
FP	0.06	0.02	1,527.90 (0.00)	0.00

Average values over 50 replicates. In each replicate there are *k* = 10 causal SNPs. Best model sizes for IHT and lasso were chosen by cross-validation. FP: false positive; TP: true positive; NA: not applicable because glmnet does not support negative binomial regression. Parentheses indicate zero-inflated Poisson regression.

### Reconstruction quality for GWAS data

Table 3 demonstrates that IHT estimates show little bias compared to estimates from lasso and marginal regressions. These trends hold with or without debiasing as described earlier. The proportion of variance explained is approximately the same in both scenarios. The displayed values are the average estimated β’s, computed among the SNPs actually found. As expected, lasso estimates show severe shrinkage compared to IHT. Estimates from marginal tests are severely overestimated because each SNP is asked to explain more trait variance than it should. As the magnitude of $\boldsymbol{\beta }_{\mathrm{true}}$ decreases, IHT estimates show an upward absolute bias, consistent with the winner’s curse phenomenon. When sample sizes are small, small effect sizes make most predictors imperceptible amid the random noise. The winner’s curse operates in this regime and cannot be eliminated by IHT. Lasso’s strong shrinkage overwhelms the bias of the winner’s curse and yields estimates smaller than true values.

**Table 3:** Comparison of coefficient estimates among IHT, lasso, and marginal regression methods

β_true_	$\beta ^{\rm Normal}_{\rm IHT}$	$\beta ^{\rm Logistic}_{\rm IHT}$	$\beta ^{\rm Poisson}_{\rm IHT}$	$\beta ^{\rm Neg Bin}_{\rm IHT}$
0.5	0.501 ± 0.015	0.508 ± 0.039	0.492 ± 0.039	0.567 ± 0.670
0.25	0.249 ± 0.013	0.256 ± 0.038	0.247 ± 0.012	0.249 ± 0.012
0.10	0.097 ± 0.014	0.125 ± 0.016	0.100 ± 0.014	0.010 ± 0.012
0.05	0.063 ± 0.007	0.108 ± 0.006	0.057 ± 0.008	0.060 ± 0.008
β_true_	$\beta ^{\rm Normal}_{\rm lasso}$	$\beta ^{\rm Logistic}_{\rm lasso}$	$\beta ^{\rm Poisson}_{\rm lasso}$	$\beta ^{\rm Neg Bin}_{\rm lasso}$
0.5	0.451 ± 0.015	0.366 ± 0.058	0.458 ± 0.037	NA
0.25	0.199 ± 0.013	0.137 ± 0.032	0.208 ± 0.015	NA
0.10	0.046 ± 0.014	0.022 ± 0.016	0.058 ± 0.016	NA
0.05	0.012 ± 0.008	0.008 ± 0.003	0.012 ± 0.009	NA
β_true_	$\beta ^{\rm Normal}_{\rm marginal}$	$\beta ^{\rm Logistic}_{\rm marginal}$	$\beta ^{\rm Poisson}_{\rm marginal}$	$\beta ^{\rm Neg Bin}_{\rm marginal}$
0.5	0.990 ± 0.500	0.983 ± 0.475	0.942 ± 0.331	0.930 ± 0.315
0.25	0.493 ± 0.189	0.480 ± 0.216	0.452 ± 0.184	0.486 ± 0.178
0.10	0.203 ± 0.078	*	0.198 ± 0.097	0.190 ± 0.090
0.05	*	*	0.165 ± 0.049	0.097 ± 0.060

Displayed coefficients are average fitted values ±1 standard error for the discovered predictors over 100 replicates. * = zero true-positive results observed on average. NA = not available because glmnet does not support negative binomial lasso regression. There are *k* = 10 true SNPs.

The results displayed in Table 3 reflect *n* = 5,000 subjects, *p* = 10,000 SNPs, 100 replicates, and a sparsity level *k* fixed at its true value *k*_true_ = 10. The λ value for lasso is chosen by cross-validation. To avoid datasets with monomorphic SNPs, the minimum MAF is set at 0.05. For linear, logistic, and Poisson regressions in marginal tests, we first screen for potential SNPs via a score test. Only top SNPs are used in the more rigorous and more computationally intensive likelihood ratio tests, which gives the β estimates. This procedure is described in Zhou et al. [38]. We ran likelihood ratio tests for all SNPs in the negative binomial model because the screening procedure is not yet implemented. However, the inflation in parameter estimates is present throughout all marginal tests.

### Correlated covariates and doubly sparse projections

Next we study how well IHT works on correlated data and whether doubly sparse projection can enhance model selection. Table 4 shows that, in the presence of extensive LD, IHT performs reasonably well even without grouping information. When grouping information is available, group IHT enhances model selection. The results displayed in Table 4 reflect *n* = 1,000 samples, *p* = 10,000 SNPs, and 100 replicates. Each SNP belongs to 1 of 500 disjoint groups containing 20 SNPs each; *j* = 5 distinct groups are each assigned 1, 2, ..., 5 causal SNPs with effect sizes randomly chosen from {−0.2, 0.2}. In all there were 15 causal SNPs. For grouped IHT, we assume perfect group information; i.e., groups containing 1–5 causative SNPs are assigned λ_*g*_ ∈ {1, 2, ..., 5}. The remaining groups are assigned λ_*g*_ = 1. As described in the Methods section, the simulated data show LD within each group, with the degree of LD between 2 SNPs decreasing as their separation increases. Although the conditions of this simulation are somewhat idealized, they mimic what might be observed if small genetic regions of whole-exome data were used with IHT.

**Table 4:** Doubly sparse IHT enhances model selection on simulated data

Model	Ungrouped IHT		Grouped IHT	
	TP	FP	TP	FP
Normal	11.1 ± 1.9	3.9 ± 1.9	12.2 ± 2.0	2.8 ± 2.0
Logistic	3.8 ± 1.6	11.2 ± 1.6	7.7 ± 2.2	7.3 ± 2.2
Poisson	11.5 ± 2.2	3.5 ± 2.2	12.4 ± 1.7	2.6 ± 1.7
Neg Bin	11.0 ± 2.1	4.0 ± 2.1	12.4 ± 1.6	2.6 ± 1.6

FP: false-positive; Neg Bin: negative binomial; TP: true positive. Results are average counts over 100 replicates ± 1 standard error. There are 15 causal SNPs in 5 groups, each containing *k* ∈ {1, 2, ...5} SNPs.

We repeated this examination of doubly sparse projection for the first 30,000 SNPs of the NFBC1966 [40] data for all samples passing the quality control measures outlined in our Methods section. We arbitrarily assembled 2 large groups with 2,000 SNPs, 5 medium groups with 500 SNPs, and 10 small groups with 100 SNPs, representing genes of different length. The remaining SNPs are lumped into a final group representing non-coding regions. In all there are 18 groups. Because group assignments are essentially random beyond choosing neighboring SNPs, this example represents the worst-case scenario of a relatively sparse marker map with undifferentiated SNP groups. We randomly selected 1 large group, 2 medium groups, and 3 small groups to contain 5, 3, and 2 causal SNPs, respectively. The non-coding region harbors 2 causal SNPs. In all there are 19 causal SNPs. Effect sizes were randomly chosen to be −0.2 or 0.2. We ran 100 independent simulation studies under this set-up, where the large, medium, small, and non-coding groups are each allowed 5, 3, 2, and 2 active SNPs. The results are displayed in Table 5. We find that even in this worst-case scenario where group information is completely lacking, grouped IHT does no worse than ungrouped IHT.

**Table 5:** Doubly sparse IHT is comparable to regular IHT on NFBC dataset using arbitrary groups

Model	Ungrouped IHT		Grouped IHT	
	TP	FP	TP	FP
Normal	17.0 ± 1.2	2.0 ± 1.2	17.0 ± 1.4	2.1 ± 1.4
Logistic	15.7 ± 1.5	3.3 ± 1.5	15.8 ± 1.6	3.2 ± 1.6
Poisson	17.1 ± 1.3	1.9 ± 1.3	17.0 ± 1.4	2.0 ± 1.4
Neg Bin	17.2 ± 1.5	1.8 ± 1.5	17.0 ± 1.5	2.1 ± 1.5

FP: false-positive; Neg Bin: negative binomial; TP: true positive. Results are average counts over 100 replicates ±1 standard error. There are 19 causal SNPs in 18 groups of various size. Simulation was carried out on the first 30,000 SNPs of the NFBC1966 [40] dataset.

### Introduction of prior weights

This section considers how scaling by prior weights helps in model selection. Table 6 compares weighted IHT reconstructions with unweighted reconstructions where all weights *w_i_* = 1. The weighted version of IHT consistently finds ∼10% more true predictors than the unweighted version. Here we simulated 50 replicates involving 1,000 subjects, 10,000 uncorrelated variants, and *k* = 10 true predictors for each GLM. For the sake of simplicity, we defined a prior weight *w_i_* = 2 for 110%all variants, including the 10 true predictors. For the remaining SNPs the prior weight is *w_i_* = 1. These choices reflect a scenario where 10% of all genotyped variants fall in protein-coding regions, including the 10 true predictors, and where such variants are twice as likely to influence a trait as those falling in non-coding regions.

**Table 6:** Weighted IHT enhances model selection

Model	Unweighted IHT		Weighted IHT	
	TP	FP	TP	FP
Normal	9.2 ± 0.4	0.8 ± 0.4	9.4 ± 0.5	0.6 ± 0.5
Logistic	7.3 ± 0.6	2.7 ± 0.6	8.0 ± 0.6	2.0 ± 0.6
Poisson	8.0 ± 0.6	2.0 ± 0.6	8.3 ± 0.6	1.7 ± 0.6
Neg Bin	9.2 ± 0.5	0.8 ± 0.5	9.4 ± 0.5	0.6 ± 0.5

FP: false-positive; Neg Bin: negative binomial; TP: true positive. Results are average counts over 100 replicates ±1 standard error. The true number of SNPs is *k* = 10.

### Hypertension GWAS in the UK Biobank

**Figure 4:**
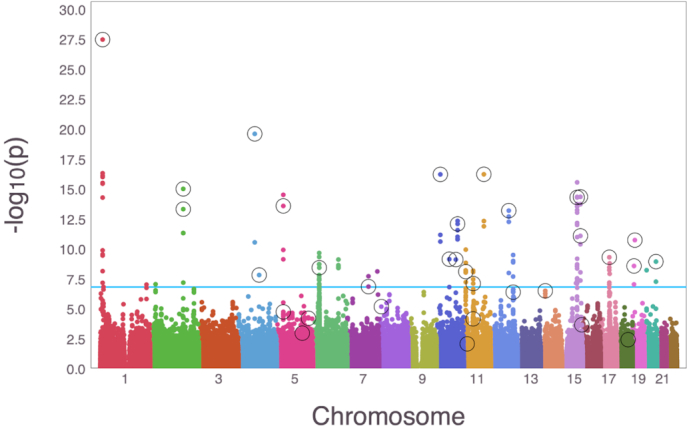
Manhattan plot comparing a logistic (univariate) GWAS vs logistic IHT on UK Biobank data. Colored dots are log_10_*P*-values from a logistic GWAS, and the circled dots are SNPs recovered by IHT.

In this section we test IHT on the second release of UK Biobank [41] data. This dataset contains ∼500,000 samples and ∼800,000 SNPs without imputation. Phenotypes are systolic blood pressure (SBP) and diastolic blood pressure (DBP), averaged over 4 or fewer readings. To adjust for ancestry and relatedness, we included the following nongenetic covariates: sex, hospital center, age, age^2^, BMI, and the top 10 principal components computed with FlashPCA2 [42]. After various quality control procedures that are outlined in the Methods section, the final dataset used in our analysis contains 185,565 samples and 470,228 SNPs. For UK Biobank analysis, we omitted debiasing, prior weighting, and doubly sparse projections.

#### Stage 2 hypertension under a logistic model

Consistent with the clinical definition for Stage 2 hypertension (S2 Hyp) [43], we designated patients as hypertensive if their SBP ≥ 140 mmHG or DBP ≥ 90 mmHG. We ran a 5-fold cross-validated logistic model across model sizes *k* = {1, 2, ..., 50}. The workload was distributed to 50 computers, each with 5 CPU cores. Each computer was assigned one model size, and each completed its task within 24 hours. The model size that minimizes thether deviance residuals is $\hat{k} = 39$. The selected predictors include the intercept, sex, age, age ^2^, BMI, the fifth principal component, and the 33 SNPs listed in Table   7. Fig.   4, generated by MendelPlots.jl [ 44], compares univariate logistic GWAS with logistic IHT. SNPs recovered by IHT are circled.

**Table 7:** UK Biobank GWAS results generated by running IHT on Stage 2 Hypertension (S2 Hyp) under a logistic model

SNP ID	Chromosome Number	Position (bp)	$\hat{\beta } \quad$	Known?
rs17367504	1	11,862,778	0.046	[45, 46]
rs757110	2	17,418,477	−0.025	
rs1898841	2	165,070,207	0.022	[46]
rs1374264	2	164,999,883	0.020	[46]
rs16998073	4	81,184,341	−0.048	[45, 46]
rs1173771	4	32,815,028	0.046	[45, 46]
rs13107325	4	103,188,709	0.030	[45, 46]
rs72742749	5	32,834,974	0.029	
rs11241955	5	127,626,884	0.028	
rs2072495	5	158,296,996	−0.027	
rs805293	6	31,688,518	−0.029	[46]
rs2392929	7	106,414,069	−0.039	[45, 46]
rs73203495	8	11,580,334	−0.031	
rs12258967	10	18,727,959	0.039	[45, 46]
rs11191580	10	104,906,211	0.039	[45, 46]
rs2274224	10	96,039,597	0.036	[46]
rs1530440	10	63,524,591	0.028	[45, 46]
rs10895001	11	100,533,021	0.043	[46]
rs2293579	11	47,440,758	−0.035	[46]
rs2923089	11	10,357,572	−0.029	[46]
rs762551	11	75,041,917	−0.027	[46]
rs4548577	11	46,998,512	0.026	
rs2681492	12	90,013,089	0.030	[45, 46]
rs10849937	12	111,792,427	0.030	[46]
rs35085068	14	23,409,909	−0.027	[46]
rs12901664	15	98,338,524	−0.027	
rs7497304	15	91,429,176	−0.021	[45, 46]
rs2677738	15	91,441,673	0.021	[46]
rs3744760	17	43,195,981	−0.043	[46]
rs292445	18	55,897,720	−0.026	
rs167479	19	11,526,765	0.036	[45, 46]
rs34328549	19	7,253,184	0.035	[46]
rs16982520	20	57,758,720	−0.030	[45, 46]

$\hat{\beta } \quad$ is the estimated effect size.

Our GitHub page records the full list of significant SNPs detected by univariate GWAS. There are 10 SNPs selected by IHT that have a *P*-value <5 × 10^−8^; 83 SNPs pass the threshold in the univariate analysis but remain unselected by IHT. IHT tends to pick the most significant SNP among a group of SNPs in LD. Table 7 shows 25 SNPs selected by IHT that were previously reported to be associated with elevated SBP/DBP [45] or that exhibit genome-wide significance when the same data are analyzed as an ordinal trait [46]. Ordinal univariate GWAS treats the different stages of hypertension as ordered categories. Ordinal GWAS has higher power than logistic or multinomial GWAS [46]. The known SNPs displayed in Table 7 tend to have larger absolute effect sizes (mean = 0.033) than the unknown SNPs (mean = 0.027). Finally, IHT is able to recover 2 pairs of highly correlated SNPs: (rs1374264, rs1898841) and (rs7497304, rs2677738) with pairwise correlations of *r*_1, 2_ = 0.59 and *r*_3, 4_ = 0.49.

### Cardiovascular GWAS in NFBC1966

We also tested IHT on data from the 1966 Northern Finland Birth Cohort (NFBC1966) [40]. Although this dataset is relatively modest with 5,402 participants and 364,590 SNPs, it has 2 virtues. First, it has been analyzed multiple times [15, 40, 47], so comparison with earlier analysis is easy. Second, due to a population bottleneck [48], the participants’ chromosomes exhibit more extensive LD than is typically found in less isolated populations. Multiple-regression methods, including the lasso, have been criticized for their inability to deal with the dependence among predictors induced by LD. Therefore, this dataset provides an interesting test case.

#### High-density lipoprotein phenotype as a normal model

Using IHT we find previously associated SNPs as well as a few new potential associations. We model the high-density lipoprotein (HDL) phenotype as normally distributed and find a best model size $\hat{k} = 9$ based on 5-fold cross-validation across model sizes *k* = {1, 2, ..., 20}. Without debiasing, the analysis was completed in 2 hours and 4 minutes with 30 CPU cores on a single machine. Table 8 displays the recovered predictors. SNP rs1800961 was replaced by rs7499892 with similar effect size if we add the debiasing step in obtaining the final model.

**Table 8:** NFBC GWAS results generated by running IHT on high-density lipoprotein (HDL) phenotype as a normal response

SNP ID	Chromosome No.	Position (bp)	$\hat{\beta } \quad$	Known?
rs6917603	6	30,125,050	0.17	[15, 45]
rs9261256	6	30,129,920	−0.07	[15]
rs9261224	6	30,121,866	−0.03	
rs7120118	11	47,242,866	−0.03	[15, 40, 45]
rs1532085	15	56,470,658	−0.04	[15, 40, 45]
rs3764261	16	55,550,825	−0.05	[15, 40, 45]
rs3852700	16	65,829,359	−0.03	
rs1800961	20	42,475,778	0.03	[45]

$\hat{\beta } \quad$ is the estimated effect size.

Importantly, IHT is able to simultaneously recover effects for SNPs (1) rs9261224, (2) rs6917603, and (3) rs6917603 with pairwise correlations of *r*_1, 2_ = 0.618, *r*_1, 3_ = 0.984, and *r*_2, 3_ = 0.62. This result is achieved without grouping of SNPs, which can further increase association power. Compared with earlier analyses of these data, we find 3 SNPs that were not listed in our previous IHT article [15], presumably due to slight algorithmic modifications. The authors of NFBC [40] found 5 SNPs associated with HDL under SNP-by-SNP testing. We did not find SNPs rs2167079 and rs255049. To date, rs255049 was replicated [47]. SNP rs2167079 has been reported to be associated with an unrelated phenotype [49].

## Discussion

Multiple-regression methods like IHT provide a principled way of model fitting and variable selection. With increasing computing power and better software, multiple-regression methods are likely to prevail over univariate methods. This article introduces a scalable implementation of IHT for GLMs. Because lasso regression can handle group and prior weights, we have also extended IHT to incorporate such prior knowledge. When this prior knowledge is available, enhanced IHT outperforms standard IHT. Given its sharper parameter estimates and more robust model selection, IHT is clearly superior to lasso selection or marginal association testing in GWAS.

Our real data analyses and simulation studies suggest that IHT can (i) recover highly correlated SNPs, (ii) avoid over-fitting, (iii) deliver better true-positive and false-positive rates than either marginal testing or lasso regression, (iv) recover unbiased regression coefficients, and (v) exploit prior information and group sparsity. Our Julia implementation of IHT exploits parallel computing strategies that scale to biobank-level data. In our opinion, the time is ripe for the genomics community to embrace multiple-regression models as a supplement to and possibly a replacement for marginal analysis.

Although we focused our attention on GWAS, the potential applications of IHT reach far beyond gene mapping. Our IHT implementation accepts arbitrary numeric data and is suitable for a variety of applied statistics problems. Genetics and the broader field of bioinformatics are blessed with rich, ultra-high-dimensional data. IHT is designed to solve such problems. By extending IHT to the realm of GLMs, it becomes possible to fit regression models with more exotic distributions than the Gaussian distributions implicit in ordinary linear regression. In our view IHT will eventually join and probably supplant lasso regression as the method of choice in GWAS and other high-dimensional regression settings.

## Methods

### Data simulation

Our simulations mimic scenarios for a range of rare and common SNPs with or without LD. Unless otherwise stated, we designate 10 SNPs to be causal with effect sizes of 0.1, 0.2, ..., 1.0.

To generate independent SNP genotypes, we first sample a MAF ρ_*j*_ ∼ Uniform(0, 0.5) for each SNP *j*. To construct the genotype of person *i* at SNP *j*, we then sample from a binomial distribution with success probability ρ_*j*_ and 2 trials. The vector of genotypes (minor-allele counts) for person *i* form row ${\bf x}_i^t$ of the design matrix **X**. To generate SNP genotypes with LD, we divide all SNPs into blocks of length 20. Within each block, we first sample *x*_1_ ∼ Bernoulli(0.5). Then we form a single haplotype block of length 20 by the following Markov chain procedure:

$$\begin{eqnarray*}
x_{i+1} = \left\lbrace \begin{array}{@{}l@{\quad }l@{}}x_i & \text{with probability } \mathit {p}\\
1 - x_i & \text{with probability } \mathit {1-p} \end{array}\right.
\end{eqnarray*}$$

with default *p* = 0.75. For each block we form a pool of 20 haplotypes using this procedure, ensuring that each of the 40 alleles (2 at each SNP) are represented at least once. For each person, the genotype vector in a block is formed by sampling 2 haplotypes with replacement from the pool and summing the number of minor alleles at each SNP.

Depending on the simulation, the number of subjects ranges from 1,000 to 120,000, and the number of independent SNPs ranges from 10,000 to 1,000,000. We simulate data under 4 GLM distributions: normal (Gaussian), Bernoulli, Poisson, and negative binomial. We generate component *y_i_* of the response vector **y** by sampling from the corresponding distribution with mean $\mu _i = g({\bf x}_i^t\boldsymbol{\beta })$, where *g* is the inverse link function. For normal models we assume unit variance, and for negative binomial models we assume 10 required failures. To avoid overflows, we clamp the mean $g({\bf x}^t_i\boldsymbol{\beta })$ to stay within [−20, 20]. (See Ad Hoc Tactics for a detailed explanation). We apply the canonical link for each distribution, except for the negative binomial, where we apply the log link.

### Real data’s quality control procedures

#### UK Biobank

Following the UK Biobank’s own quality control procedures, we first filtered all samples for sex discordance and high heterozygosity/missingness. Second, we included only participants of European ancestry and excluded first- and second-degree relatives on the basis of empiric kinship coefficients. Third, we also exclude impuded participants who had taken hypertension-related medications at baseline. Finally, we only included participants with $\ge 98\%$ genotyping success rate over all chromosomes and SNPs with $\ge 99\%$ genotyping success rate over all included individuals. Calculation of kinship coefficients and filtering were carried out via the OpenMendel module SnpArrays [50]. Remaining missing genotypes were imputed using modal genotypes at each SNP. After these quality control procedures, our UK Biobank data are the same data that were used by German et al. [46].

#### Northern Finland Birth Cohort

We imputed missing genotypes with Mendel [51]. Following Keys et al. [15], we excluded participants with missing phenotypes, fasting participants, and participants receiving diabetes medication. We conducted quality control measures using the OpenMendel module SnpArrays [50]. On the basis of these measures, we excluded SNPs with MAF ≤ 0.01 and Hardy-Weinberg equilibrium *P*-values ≤10^−5^. Concerning non-genetic predictors, we included sex (the sexOCPG factor defined in Sabatti et al. [40]) as well as the first 2 principal components of the genotype matrix computed via PLINK 2.0 alpha [52]. To put predictors, genetic and non-genetic, on an equal footing, we standardized all predictors to have mean zero and unit variance.

### Linear algebra with compressed genotype files

The genotype count matrix stores minor-allele counts. The PLINK genotype compression protocol [52] compactly stores the corresponding 0’s, 1’s, and 2’s in 2 bits per SNP, achieving a compression ratio of 32:1 compared with storage as floating point numbers. For a sparsity level *k* model, we use OpenBLAS (a highly optimized linear algebra library) to compute predicted values. This requires transforming the *k* pertinent columns of **X** into a floating point matrix **X**_*k*_ and multiplying it by the corresponding entries $\boldsymbol{\beta }_k$ of $\boldsymbol{\beta }$. The inverse link is then applied to ${\bf X}_k\boldsymbol{\beta }_k$ to give the mean vector $\boldsymbol{\mu }=g({\bf X}_k\boldsymbol{\beta }_k)$. In computing the GLM gradient (formula 3), formation of the vector ${\bf W}_1({\bf y}-\boldsymbol{\mu })$ involves no matrix multiplications. Computation of the gradient ${\bf X}^t{\bf W}_1({\bf y}-\boldsymbol{\mu })$ is more complicated because the full matrix **X** can no longer be avoided. Fortunately, the OpenMendel module SnpArrays [50] can be invoked to perform compressed matrix times vector multiplication. Calculation of the step length of IHT requires computation of the quadratic form $\nabla L(\boldsymbol{\beta }_n)^t {\bf X}^t {\bf W}_2 {\bf X} \nabla L(\boldsymbol{\beta }_n)$. Given the gradient, this computation requires a single compressed matrix times vector multiplication. Finally, good statistical practice calls for standardizing covariates. To standardize the genotype counts for SNP *j*, we estimate its MAF *p_j_* and then substitute the ratio $\left(x_{ij}-2p_j\right)/\left[2p_j(1-p_j)\right]^{1/2}$ for the genotype count *x_ij_* for person *i* at SNP *j*. This procedure is predicated on a binomial distribution for the count *x_ij_*. Our previous article [15] shows how to accommodate standardization in the matrix operations of IHT without actually forming or storing the standardized matrix.

Although multiplication via the OpenMendel module SnpArrays [50] is slower than OpenBLAS multiplication on small datasets, it can be as much as 10 times faster on large datasets. OpenBLAS has advantages in parallelization, but it requires floating point arrays. Once the genotype matrix **X** exceeds the memory available in RAM, expensive data swapping between RAM and disk memory sets in. This dramatically slows matrix multiplication. SnpArrays is less vulnerable to this hazard owing to compression. Once compressed data exceed RAM, SnpArrays also succumbs to the swapping problem. Current laptop and desktop computers seldom have >32 GB of RAM, so we may wish to resort to cluster or cloud computing when input files exceed 32 GB.

### Computations involving non-genetic covariates

Non-genetic covariates are stored as double or single precision floating point entries in an *n* × *r* design matrix **Z**. To accommodate an intercept, the first column should be a vector of 1’s. Let $\boldsymbol{\gamma }$ denote the *r* vector of regression coefficients corresponding to **Z**. The full design matrix is the block matrix $({\bf X} \, {\bf Z})$. Matrix multiplications involving $({\bf X} \, {\bf Z})$ should be carried out via

$$\begin{eqnarray*}
({\bf X} \, {\bf Z}){\begin{pmatrix}\boldsymbol{\beta } \\
\boldsymbol{\gamma } \end{pmatrix}} & = & {\bf X} \boldsymbol{\beta } + {\bf Z} \boldsymbol{\gamma } \quad \text{and} \quad ({\bf X} \, {\bf Z})^t \, {\bf v} = {\begin{pmatrix}\bf X^t{\bf v} \\
{\bf Z}^t {\bf v} \end{pmatrix}}.
\end{eqnarray*}$$

Adherence to these rules ensures a low memory footprint. Multiplication involving **X** can be conducted as previously explained. Multiplication involving **Z** can revert to BLAS.

### Parallel computation

The OpenBLAS library accessed by Julia is inherently parallel. Beyond that we incorporate parallel processing in cross-validation. Recall that in *q*-fold cross-validation we separate subjects into *q* disjoint subsets. We then fit a training model using *q* − 1 of those subsets on all desired sparsity levels and record the mean-squared prediction error on the omitted subset. Each of the *q* subsets serves as the testing set exactly once. Testing error is averaged across the different folds for each sparsity level *k*. The lowest average testing error determines the recommended sparsity.

MendelIHT.jl offers 2 parallelism strategies in cross-validation. Either the *q* training sets are each loaded to *q* different CPUs where each computes and tests different sparsity levels sequentially, or each of the *q* training sets is cycled through sequentially and each sparsity parameter is fitted and tested in parallel. The former tactic requires enough disk space and RAM to store *q* different training datasets [where each typically requires (*q* − 1)/*q* GB of the full data] but offers immense parallel power because one can assign different computers to handle different sparsity levels. This tactic allows one to fit biobank-scale data in less than one day, assuming enough storage space and computers are available. The latter tactic requires cycling through the training sets sequentially. Because intermediate data can be deleted, this tactic only requires enough disk space and RAM to store one copy of the training set. MendelIHT.jl uses one of Julia’s [22] standard libraries, Distributed.jl, to achieve the aforementioned parallel strategies.

### Ad hoc tactics to prevent overflows

In Poisson and negative binomial regressions, the inverse link argument $\exp ({\bf x}_i^t\boldsymbol{\beta })$ experiences numerical overflows when the inner product ${\bf x}_i^t\boldsymbol{\beta }$ is too large. In general, we avoid running Poisson regression when response means are large. In this regime a normal approximation is preferred. As a safety feature, MendelIHT.jl clamps values of ${\bf x}_i^t\boldsymbol{\beta }$ to the interval [−20, 20]. Note that penalized regression is hindered by the same overflow catastrophes.

### Convergence and backtracking

For each proposed IHT step we check whether the objective $L(\boldsymbol{\beta })$ increases. When it does not, we step-halve at most 5 times to restore the ascent property. Convergence is declared when

$$\begin{eqnarray*}
\frac{||\boldsymbol{\beta }_{n+1} - \boldsymbol{\beta }_n||_\infty }{||\boldsymbol{\beta }_n||_\infty + 1} \lt \rm{Tolerance},
\end{eqnarray*}$$

with the default tolerance being 0.0001. The addition of 1 in the denominator of the convergence criterion guards against division by 0.

## Availability of Source Code and Requirements

Project name: MendelIHT

Project home page: https://github.com/OpenMendel/MendelIHT.jl

Operating systems: Mac OS, Linux, Windows

Programming language: Julia 1.0, 1.2

License: MIT

RRID:SCR_018292

bio.tools ID: bio.tools/mendeliht.jl

The code to generate simulated data, as well as their subsequent analysis, is available in our GitHub repository under the "figures" folder. Project.toml and Manifest.toml files can be used together to instantiate the same computing environment in our article. Notably, MendelIHT.jl interfaces with the OpenMendel [38] package SnpArrays.jl [50] and JuliaStats’s packages Distribution.jl [53] and GLM.jl [54].

## Availability of Supporting Data and Materials

The Northern Finland Birth Cohort 1966 (NFBC1966) [40] was downloaded from dbGaP under dataset accession pht002005.v1.p1. UK Biobank data are retrieved under Project ID: 48152 and 15678. An archival snapshot of the code and other supporting data is available via the *GigaScience* Database, GigaDB [55].

## Additional Files

**Supplementary information**: Supplementary data are available in GigaDB.

giaa044_GIGA-D-19-00398_Original_Submission

giaa044_GIGA-D-19-00398_Revision_1

giaa044_GIGA-D-19-00398_Revision_2

giaa044_Response_to_Reviewer_Comments_Original_Submission

giaa044_Response_to_Reviewer_Comments_Revision_1

giaa044_Reviewer_1_Report_Original_Submission Jian Zeng -- 12/15/2019 Reviewed

giaa044_Reviewer_1_Report_Revision_1 Jian Zeng -- 3/4/2020 Reviewed

## Abbreviations

BLAS: Basic Linear Algebra Subprograms; BMI: body mass index; bp: base pairs; CPU: central processing unit; DBP: diastolic blood pressure; GLM: generalized linear models; GWAS: genome-wide association studies; HDL: high-density lipoprotein; HTP: hard thresholding pursuit; IHT: iterative hard threhsolding; LD: linkage disequilibrium; LDL: low-density lipoprotein; MAF: minor-allele frequency; MCP: minimax concave penalty; Neg Bin: negative binomial; NFBC: Northern Finland Birth Cohort; NHLBI: National Heart, Lung, and Blood Institute; NIH: National Institutes of Health; NIHT: normalized iterative hard threshold algorithm; RAM: random access memory; S2 Hyp: Stage 2 hypertension; SBP: systolic blood pressure; SNP: single-nucleotide polymorphism.

## Competing Interests

The authors declare that they have no competing interests.

## Ethics and Consent for Publication

As described in [40], informed consent from all study pardataticipants of NFBC1966 was obtained using protocols approved by the Ethical Committee of the Northern Ostrobothnia Hospital District.

## Funding

B.B.C. was supported by NIH T32-HG002536 training grant and the 2018 Google Summer of Code. K.L.K. was supported by a diversity supplement to NHLBI grant R01HL135156, the UCSF Bakar Computational Health Sciences Institute, the Gordon and Betty Moore Foundation grant GBMF3834, and the Alfred P. Sloan Foundation grant 2013-10-27 to UC Berkeley through the Moore-Sloan Data Sciences Environment initiative at the Berkeley Institute for Data Science (BIDS). E.M.S, K.L., and H.Z. were supported by grants from the National Human Genome Research Institute (HG006139) and the National Institute of General Medical Sciences (GM053275). J.S.S. was supported by grants from the National Institute of General Medical Sciences (GM053275), the National Human Genome Research Institute (HG009120), and the National Science Foundation (DMS-1264153). C.A.G. was supported by the Burroughs Wellcome Fund Inter-school Training Program in Chronic Diseases (BWF-CHIP).

## Authors' Contributions

B.B.C., K.L.K., E.M.S., J.S.S., and K.L. contributed to the design of the study, interpretation of results, and writing of the original draft manuscript. B.B.C. designed and implemented the simulations and conducted the data analyses. C.A.G., H.Z., and J.J.Z. contributed to the analysis of UK Biobank results. B.B.C. and K.L.K. developed the software. B.B.C. and K.L. developed the algorithms. E.M.S. assisted in the comparisons to marginal GWAS. All authors have read, made suggestions, and ultimately approved the final manuscript.
