# Supplementary material for: Iterative hard thresholding in genome-wide association studies: Generalized linear models, prior weights, and double sparsity
Source: Gigascience. 2020 Jun 3;9(6):giaa044. doi: 10.1093/gigascience/giaa044 (PMC7268817; doi:10.1093/gigascience/giaa044)
Supplement: giaa044_Response_to_Reviewer_Comments_Original_Submission [file giaa044_response_to_reviewer_comments_original_submission.pdf]

Author's Response To Reviewer Comments

Close

Our detailed responses to our reviewers are attached as a PDF in this revision.

Close
