# Supplementary material for: Iterative hard thresholding in genome-wide association studies: Generalized linear models, prior weights, and double sparsity
Source: Gigascience. 2020 Jun 3;9(6):giaa044. doi: 10.1093/gigascience/giaa044 (PMC7268817; doi:10.1093/gigascience/giaa044)
Supplement: giaa044_Reviewer_1_Report_Original_Submission — Jian Zeng -- 12/15/2019 Reviewed [file giaa044_reviewer_1_report_original_submission.pdf]

Reviewer Report

Title: Iterative Hard Thresholding in GWAS: Generalized Linear Models, Prior Weights, and Double Sparsity

Version: Original Submission **Date:** 12/15/2019

Reviewer name: Jian Zeng

Reviewer Comments to Author:

I would like to thank the authors for responding each of my comments. Based on their revision, I have following follow-up comments.

1. The authors explain that the inflation in the estimation of small causal effects in Table 3 is due to the winner's curse and inadequate shrinkage imposed by IHT. Lasso does not seem to suffer the winner's curse problem but introduces too much shrinkage toward zero. I think it would be useful to also show the beta estimate from the standard GWAS model to demonstrate that IHT (should) results in relatively smaller bias comparing to the GWAS model with stringent genome-wide significant threshold.
2. The authors add the results of false positives in Tables 4-6. First, because the total number of causal variants are not the same between scenarios, I still think it is more informative and straightforward to show the rate rather than the actual numbers. Second, if I read it correctly, what they compute for the false positives is the proportion of false positives among all rejections of the null, a kind of false discovery rate (FDR). Have they looked at the type I error rate under the null model, which is what typically controlled in GWAS? Finally, it is not clear how they control the FDR and the FDR in their simulations seems a bit too high (> 0.05).
3. The authors show the results of the UK Biobank (UKB) data analysis using IHT. It is important to show any clear advantage of their method under a fair comparison with the traditional GWAS. That is, they should compare their results to the traditional GWAS with p-values and LD clumping using the same UKB samples. They could perform the GWAS analysis themselves to identify nearly independent loci or compare to the published GWAS using the same UKB sample size. The results they choose to compare to is from GWAS catalog 2016, which is obviously out-dated.
4. In the response to reviewer 2's second comment, the authors say that 'the performance of group-IHT is a lower bound for doubly sparse IHT after our modification'. I presume the group-IHT they mention is IHT with variable selection for groups but not within a group. Is the statement their speculation or do they have some results to show that? They say they have incorporated this into the revised article but I couldn't find much text about it. Apart from the comment itself, I hope the authors can highlight the changes in the revised manuscript and point to the changes regarding to each comment.

Level of Interest

Please indicate how interesting you found the manuscript: Choose an item.

Quality of Written English

Please indicate the quality of language in the manuscript: Choose an item.

Declaration of Competing Interests

Please complete a declaration of competing interests, considering the following questions:

- Have you in the past five years received reimbursements, fees, funding, or salary from an organisation that may in any way gain or lose financially from the publication of this manuscript, either now or in the future?
- Do you hold any stocks or shares in an organisation that may in any way gain or lose financially from the publication of this manuscript, either now or in the future?
- Do you hold or are you currently applying for any patents relating to the content of the manuscript?
- Have you received reimbursements, fees, funding, or salary from an organization that holds or has applied for patents relating to the content of the manuscript?
- Do you have any other financial competing interests?
- Do you have any non-financial competing interests in relation to this paper?

If you can answer no to all of the above, write 'I declare that I have no competing interests' below. If your reply is yes to any, please give details below.

I declare that I have no competing interests

I agree to the open peer review policy of the journal. I understand that my name will be included on my report to the authors and, if the manuscript is accepted for publication, my named report including any attachments I upload will be posted on the website along with the authors' responses. I agree for my report to be made available under an Open Access Creative Commons CC-BY license (http://creativecommons.org/licenses/by/4.0/). I understand that any comments which I do not wish to be included in my named report can be included as confidential comments to the editors, which will not be published.

Choose an item.

To further support our reviewers, we have joined with Publons, where you can gain additional credit to further highlight your hard work (see: https://publons.com/journal/530/gigascience). On publication of this paper, your review will be automatically added to Publons, you can then choose whether or not to claim your Publons credit. I understand this statement.

Yes Choose an item.
